# TLR Specific Immune Responses against Helminth Infections

**DOI:** 10.1155/2017/6865789

**Published:** 2017-10-31

**Authors:** Sivaprakasam Rajasekaran, Rajamanickam Anuradha, Ramalingam Bethunaickan

**Affiliations:** ^1^Department of Immunology, National Institute for Research in Tuberculosis, Chennai, India; ^2^International Center for Excellence in Research, National Institutes of Health, National Institute for Research in Tuberculosis, Chennai, India

## Abstract

Despite marked improvement in the quality of lives across the globe, more than 2 million individuals in socioeconomically disadvantaged environments remain infected by helminth (worm) parasites. Owing to the longevity of the worms and paucity of immunologic controls, these parasites survive for long periods within the bloodstream, lymphatics, and gastrointestinal tract resulting in pathologic conditions such as anemia, cirrhosis, and lymphatic filariasis. Despite infection, an asymptomatic state may be maintained by the host immunoregulatory environment, which involves multiple levels of regulatory cells and cytokines; a breakdown of this regulation is observed in pathological disease. The role of TLR expression and function in relation to intracellular parasites has been documented but limited studies are available for multicellular helminth parasites. In this review, we discuss the unique and shared host effector mechanisms elicited by systemic helminth parasites and their derived products, including the role of TLRs and sphingolipids. Understanding and exploiting the interactions between these parasites and the host regulatory network are likely to highlight new strategies to control both infectious and immunological diseases.

## 1. Introduction

Helminth parasites (worms) include an array of metazoan organisms. Over 60% of the world populations are at the risk of helminth infections in tropical and subtropical regions [1]. Parasitic infections are major public health problems that have impact on socioeconomic influence. Chronic infection may lead to physical disabilities, anemia, and malnourishment [2]. Helminth parasitic infection has been largely eliminated in developed countries due to control of the insect vector population by the safe disposal of human waste and the availability of efficient drugs. Nevertheless, in developing countries, these types of parasite control methods are often not yet practical and helminths persist as a significant biomedical problem.

Helminth parasites have evolved to survive and reproduce within immune-exposed niches, such as the blood, lymphatics, and gastrointestinal tract [2, 3]. Several parasites have a complex multistage lifecycle, which requires numerous intermediate hosts for completion. Inside the mammalian host, parasites undergo extensive growth and differentiation to produce developmental stages ready for transmission to the next intermediate host. Larva migrates within the host to its suitable niche that supports its growth and reproduction. The resulting offspring are capable of transmission from one host to another; this process varies among helminths [4]. The social and medical impact of the global parasitic worm burden necessitates more attention and research focus on modulation of immune responses to helminth infection and factors that influence disease pathogenesis [5]. The host immune response to helminths includes multiple strategies for induction of regulatory networks and immune responses that involve both the innate and adaptive immune system [6]. Helminth parasites have evolved immune evasion strategies necessary for their continued transmission. This immune evasion is achieved at the expense of both antigen-presenting cells (APCs) and T cells. Similar to intracellular parasitic infections, pattern-recognition receptors (PRR) play a pivotal role in initiating the host immune response against multicellular helminth parasites [6]. Most of the pathogen-associated molecular patterns (PAMPs) from these parasites are recognized by Toll-like receptors (TLRs) [7]. TLRs are expressed on many cell types, for example, epithelial cells of the gastrointestinal and respiratory tracts, myofibroblasts, enteroendocrine cells, astrocytes, and immune cells such as T cells, B cells, and dendritic cells (DCs) [8]. TLRs dictate the downstream pathways involved in adaptive immune responses by influencing multiple antigen-presenting cell (APC) functions [9]. The potential contribution of TLRs to fighting parasitic infections has gained much attention in the last decade [10]. In addition to TLR, NOD (nucleotide oligomerization domain-like receptor) recognizes intracellular PAMPS and initiates signaling pathways that induce production of inflammatory cytokines [11, 12].

## 2. Overview of TLR

TLRs are central players in many aspects of microbial elimination, including recruitment of phagocytes to infected tissue, following microbial killing. TLRs are expressed by macrophages and dendritic cells (DCs); T and B lymphocytes also express TLRs [13]. TLRs are membrane spanning and noncatalytic receptors, which are capable of recognizing structurally conserved molecules derived from pathogens and directing the downstream immune response [14]. Currently thirteen TLRs have been identified, TLR1–TLR13; of these, TLR1–TLR9 are conserved between humans and mice. In mice TLR10 is nonfunctional due to a retrovirus insertion whereas TLR11–TLR13 are present within endosomal compartments of mice but are lost from the human genome [15]. TLR1, TLR2, TLR4, TLR5, and TLR6 are expressed on the plasma membrane and TLR3, TLR7, TLR8, and TLR9 are present in endosome of leukocytes. These receptors are expressed on various immune and nonimmune cells in a variety of combinations in order to recognize most of the pathogen-associated molecular patterns (PAMPs), thereby providing a link between the innate and adaptive immune systems [16, 17]. TLR signals through MyD88 pathway leading to activation of MAPK and induces the translocation of nuclear factor kappa B (NF-kB) to the nucleus. NF-kB promotes the transcription and synthesis of proinflammatory cytokines [18].

## 3. TLR Pathway

Immune and nonimmune cells with unique combinations of TLR expression patterns have been identified within mammals [8]. They can recognize PAMPs derived from parasites or microbes, including proteins, lipoproteins, lipids, and nucleic acids. In addition, endogenous ligands (heat-shock proteins, fragments from extracellular matrix, fibrinogen, and end products of cellular apoptosis like DNA and RNA) can also bind to TLRs and trigger inflammatory cascades [19]. TLRs are important in the recognition of* Leishmania* species [20]. They sequentially trigger the innate and then adaptive immune responses required for controlling* Leishmania* parasite [21]. Purified* Leishmania* lipophosphoglycan stimulates upregulation of TLR2 on human NK cells and elicits leishmanicidal reactions via release of inflammatory mediators, for example, TNF-*α*, IFN-*γ*, nitric oxide (NO), and reactive oxygen species (Th1 response). TLR2 can also induce the antileishmanial immune response through decreased expression of TLR9. The PAMP-dependent DC activation could be based on TLR expression. Plasmacytoid dendritic cells (PDC) express TLR9 and recognize CpG motifs in mice while myeloid DC (mDC) expresses TLR4 and its reacts to LPS [22]. Endogenous RNA and DNA activate TLR7 and TLR9, by entering into the endosomal compartment, thereby inducing production of proinflammatory cytokines by plasmacytoid (pDC) and conventional DCs (cDC) [23]. Endogenous TLRs have been crucial for resistance to* Leishmania major* [18]. Within mammalian genomes, the CpG motif occurs much less frequently and remains highly methylated; as a result, TLR9 does have a limited activation role in eliciting innate immunity. CpG DNA induces a conformational change in TLR9 that is required for its activation [24]. TLR9 recognizes unmethylated CpG motifs as a conserved molecular pattern in pathogen DNA and abnormal composition, structure, or chemical features in any kind of DNA [25]. TLR9 signaling is essential for NK cell activation and production of IL-12 by bone marrow-derived DC, which can reduce the parasite burden [10, 26]. Fakher et al. have shown that TLR9-deficient mice have increased* Leishmania* burden which indicates that TLR9 plays an important role in reducing parasite burden [27]. Steady-state production of IL-12 by migratory CD103(+) DCs, independent of signals from commensals or TLR-initiated events, was necessary and sufficient to exert the suppressive effects on Th2 response development in* S. mansoni* [28].

Downregulation of TLRs is a strategy used by protozoa to evade immune responses. Protozoan parasites such as* Trypanosome* spp. and* Entamoeba histolytica* were shown to inhibit the immune response particularly, by downregulating TLR2 expression [29]. Similarly, mRNA expression of TLR3, TLR4, TLR5, and TLR7 from monocyte derived DC was significantly downregulated by live microfilariae (Mf) of* B. malayi* (BmA) [30]. Filarial infected individuals have shown decreased mRNA and protein expression of TLR1, TLR2, TLR4, and TLR9 in B cells [31]. T cells play an additional role in TLR signaling, because T cells express many of the TLRs. In lymphatic filarial infected patients, T cells express lower levels of TLR1, TLR3, and TLR4 after stimulation by B cells and monocytes [32].

TLR plays the major role in intestinal homeostasis [33]. The MUC2 genes are associated with TLR pathways. Helminth and it products may stimulate physical barrier function of IECs by TLR [34]. Control of inflammation by helminths in the TLR pathway is highly possible for efficient host protection through TLR-dependent proinflammatory cascades elicited by parasitic infections, which must be firmly controlled to evade severe pathology, reviewed by [35].

## 4. Immune Response to Helminths

Innate and adaptive immune systems are crucial for the induction of type 2 immunity, which distinguishes the response to helminth infection. The key players in T helper (Th) type 2 immunity are CD4^+^ Th2 cells and involve the cytokines IL-4, IL-5, IL-9, IL-10, and IL-13 and immunoglobulin (Ig)E. Th2-type immune responses are comprised of three features: inflammation, wound repair, and resistance to helminths [36]. A diverse range of multicellular parasites dwelling inside humans elicit a stereotypical immune response in order to protect themselves from immune attack [6, 37, 38]. Helminth influence of DC may also be facilitated by method by the enzymatic activities of helminth-derived products. For example, helminth parasites inhibit the host innate immune response, initially by releasing many types of enzymatically active products which are assumed to play a key role in determining and supporting infection by contributing to the degrading the soluble antiparasitic molecules or the weakening of innate immune cells [39]. This results in the production of Th2 associated cytokines, particularly, IL-5, IL-13, and IL-4 together with IgE elicited through mast cell and eosinophil mobilization. Th2 immune responses are not however sufficient to expel the parasite [40]. IL-4 and IL-13 together with apoptotic cells provoke host protection against helminth infections and the anti-inflammatory and tissue repair phenotype in macrophages [41, 42]. Several animal studies, carried out with* Schistosoma mansoni*, have shown that parasites are capable of attenuating Th1 responses (decreased IFN-*γ*, TNF-*α*, IL-12, and NO) and promoting Th2 immune responses (IL-10 and TGF-*β*) [43, 44]. The filarial endosymbiont* Wolbachia* is known to elicit immune responses through TLR2 and TLR4 and is known to be the major mediator of inflammatory responses in lymphatic filariasis and onchocerciasis [45, 46]. Immunologists are often intrigued by the way the host tolerates helminth infection by the immunomodulation, even after exhibiting severe immunopathological condition [47]. Gao et al. suggested that TLR4 might play a role in the protection against infection, whereas TLR2 was favorable for the parasite [48]. The expression of TLR2 and subsequently NF-kB was decreased in intestinal schistosomiasis 12 weeks after infection even though the parasite burden is still high [49]. TLR-related genes are generally decreased during the course of* Schistosoma* infection. TLR1, TLR3, TLR7, and TLR8 are strongly repressed, with the appearance of the eggs at week 8 after infection, and TLR3 shows most repression [50].

## 5. Helminth Immune Modulation through TLR

Inflammatory signal from TLR is a defensive degree of the host body to warrant elimination of harmful extortions posed by infectious agents as well as fastening the healing process. Conversely, the Th1 influenced inflammatory consequences orchestrated by TLRs also engage in destroying pathogenic infections but also can provoke decisive pathological effects [16]. Likewise, pathogen modified TLR signaling progresses to Th2 related response favorable for the pathogen that will lead disease progression [34]. Hence, a sufficient stability between pro- and anti-inflammatory immune responses is of massive significance to restore the normal physiological conditions of the host body during and after a pathogenic infection [40]. Wang et al. isolated immunomodulatory peptide called SJMHE1 from the HSP60 protein of* Schistosoma japonicum* and showed that small molecule peptide that has progressed during host-parasite interactions is of huge significance in the search for novel anti‐inflammatory agents and therapeutic goals for autoimmune diseases [51, 52].

TLRs trigger an intracellular signaling cascade through Toll/interleukin-1 receptor (TIR) and through the recruitment of adaptor molecules, such as TICAM-1, MyD88 [53], TRIF, and TRAM [54, 55]. These adaptor molecules act either independently or in combination, to induce transcription factors such as c-Jun-N-terminal kinases (JNK), mitogen-activated protein kinases (MAPK), p38, extracellular signal-regulated kinase (ERK), and NF-kB (nuclear factor kappa B), leading to the transcription of inflammatory and immunomodulatory genes including costimulatory molecules, cytokines, and chemokines [56–58]. Ongoing infections in the deficiency of certain TLR diverge adaptive responses, which aggravates the immunopathology of the host, which has been shown by various studies [35, 39]. More recently, the way TLR mediates interaction between these multicellular parasites and the host immune system has been well documented. During acute phase of helminth infections, DC promotes Th1 environment through the activation of TLR, which would match with induced Th1 responses [6, 38, 40]. Helminth antigen contains proteins, glycoproteins, and glycolipids. DC induces proinflammatory activation and maturation due to impassive behaviour on activation and the failure with helminth antigens. The calreticulin protein isolated from* Heligmosomoides polygyrus* can be able to provoke IL-4 secretion through triggering class A scavenger receptor [59]. Immunomodulatory activity by ES of different species is well characterized in the nematode* Heligmosomoides polygyrus* [60] and the trematode* Fasciola hepatica* [61].* Acanthocheilonema viteae ES-62* product contains glycoprotein, which triggers TLR4 in turn inducing Th2 type of immune response which in turn determines the phenotype of the APCs [62–64]. However, studies carried out on innate cells with* Schistosoma mansoni's* soluble egg antigen (SEA) were not capable of eliciting such TLR response, instead dampening the release of proinflammatory cytokines with response to LPS [65, 66]. Several studies have shown that the inhibitory consequences of helminth-derived factors on TLR stimulated triggering as determined by proinflammatory cytokine secretion and expression of costimulatory molecules [67, 68].

Schistosome soluble egg antigen (SEA) and ES products freed by the egg stage of the parasite encompass effective Th2-inducing and immunomodulatory activity. SEA from* Schistosoma mansoni* was shown to be a tremendously strong inducer of Th2 responses, even in the absence of current infection or any supplement of adjuvant.* Schistosoma mansoni* also expresses glycans that have been shown to reveal immunomodulatory functions [69]. Schistosomal infection promotes the differentiation of DC and secretes IL10, thereby inducing Tregs, mediated through the downstream effect of TLR2 [64]. TLR2 is a receptor that plays an important role in filarial infection; the filarial endosymbiont* Wolbachia* is known to elicit immune responses through TLR2 and TLR4 and is known to be the major mediator of inflammatory responses in lymphatic filariasis and onchocerciasis [45, 46]. Studies carried out by [45] have revealed that, upon harboring* Wolbachia*, an endosymbiont within the filarial parasite, it can interact with the innate immune system through TLR2 and TLR4. Our own studies have shown that humans infected with filarial infection revealed a tardy response against APC and T cell specific TLR1, TLR2, TLR4, TLR9, and their ligands, expressing decreased proinflammatory cytokines [31, 32]. Thus, most of these infections impair the Th1 response, mainly through impairment of conventional DC maturation, and favor Th2 or regulatory immune response. Most of the helminth derivatives, including phosphorylcholine containing glycoprotein ES-62, induce anti-inflammatory or Th2 response in* Acanthocheilonema viteae* infection [62, 63, 70]. This nematode is of particular interest and it does not contain the endosymbiont bacteria* Wolbachia*. This relationship is essential to other filarial worms since its death through antibiotic treatment leads to worm sterility and death. The bacteria are also thought to mediate immune responses by triggering TLR2 and TLR4 [45]. Phospholipids from schistosomes and* Ascaris* worms also trigger TLR2 and the lysophosphatidyl serine could activate DCs to induce Th2 and IL-10-producing Treg. Several studies have demonstrated that ongoing infections in the absence of certain TLR deviates adaptive responses, which exacerbates the immunopathology of the host, reviewed in [71]. Initiation of alternatively activated macrophages (AAM) was expendable for the defending effect of* Litomosoides sigmodontis* infection on* E. coli*-provoked peritoneal sepsis, whereas TLR2-activation during the reprogramming of functional macrophages was crucial [72]. Fatty acid binding protein (FABP) plays an important role in parasite nutrition [61].* Fasciola hepatica* fatty acid binding protein (Fh12) blocks induction of inflammatory mediators* in vitro* and* in vivo* and in doing so completely inhibits activation of TLR4 by LPS in a dose-dependent manner [73]. Nullification of the ES-62-mediated suppression of LPS leads to the production of IL-6, IL-12p70, and TNF-*α* by DCs. Thus, by exploiting this homeostatic regulatory mechanism, ES-62 can protect against abnormal inflammation, can support parasite survival, and disclose therapeutic potential in inflammatory disease [74].

Sustained infection or impairment of innate immune cells occurs due to degradation of antiparasitic molecules determined by the helminth parasites, which present inside the host [39]. Parasite survival is promoted in the host; by downregulation of an antigen specific T cell proliferation [75–77] ES-62 from rodent filarial nematode inhibit the activation of B and T cell [70]. Parasite derived molecules from* Schistosoma* are processed through TLR4 and MyD88 dependent pathway [78].* Schistosoma* egg product primes DC to drive Th2 responses [39] and LFNP III stimulates IL-10 producing B1 cells in mice [79]. Rodent malarial secretory product induces immunomodulation by inhibition of B and T lymphocyte proliferation and inhibition of maturation of naïve DCs priming T cells and inhibition of IFN-*γ*, IL-12, and IL-17. Parasite derived lipids signal through TLR2 [80].

## 6. TLR Signaling

TLR signaling has been extensively investigated since 1999. The cytoplasmic domain of TLR, termed Toll/interleukin-1 receptor (TIR), is highly conserved and functions as binding site for downstream adaptor molecules. Signaling by TLRs involves a variety of adaptor proteins [81], the most common one being the myeloid differentiation marker 88 (MyD88), used by all TLRs, except TLR3 [82]. Downstream targets of MyD88 include nuclear factor kappa B (NF-*κ*B), mitogen-activated protein kinases (MAPKs) (p38, JNK, and ERK1/2), KB kinase inhibitor (IKK), and interferon regulatory factors (IRF) [83]. After endocytosis into endosomes, both TLR3 and TLR4 induce IFN-*β* by downstream signaling mediated by the alternate adapter TRIF. TLR2 and TLR4 require use of TIRAP in addition to MyD88 for downstream signaling [84, 85]. MyD88−/− mice infected by* T. gondii* showed diminished IL-12 levels and Th1 cell responses.

TIR domain is a key molecular module of TLR mediated innate immune response pathways. All mammalian TLRs contain TIR domains in their C-terminal regions. Homo- or heterotypic dimerization of TIR domains is required to initiate downstream signaling. Similar to most of the microbes, helminth parasites evade host immune response by dampening TLR expression and downregulating the TLR mediated cell signaling [86], whereas helminth-derived molecules are capable of activating TLRs through a set of kinases, resulting in Th1 type of immune response. The nuclear factor kappa B (NF-kB) pathway activated by triggering TLRs, as a result of induction of inflammatory responses occurs. NF-kB pathway and interferon regulatory factor (IRF) pathway receive signals from activated Toll/IL-1R (TIR) domain and start signal through five different adaptor molecules. by binding with specific ligand and contact with the ligand, TLR recruits an adaptor protein either to TIR domain or with IL-1 receptor associated kinases (IRAKs) [87]. IRAKs have important role in the early stages of TLR signaling. MyD88 interacts with IRAK1 and subsequently recruits IRAK1 or IRAK2 [88].

MyD88 involves all TLR signaling pathways except TLR3. MyD88 binds with MyD88 adaptor-like (MAL) protein (MAL). MyD88 independent pathway is activated through binding of TLR3 to its adaptor molecule TIR-related adaptor protein inducing interferon (TRIF). TLR4 signals through TRIF binding to its adaptor TRIF-related adaptor molecule (TRAM) and also through MAL/MyD88 protein complex. It has been shown that TLR4 ligands like LPS are capable of strongly activating JNK, MAP-kinase, and ERK, whereas molecule like LNFPIII can phosphorylate only ERK. Besides, TLR4 can be activated through MyD88-independent pathway by interferons through TRAM/TRIF complex [58, 89, 90] NF-kB comprised p50, p65, p52, RelB, and c-Rel subunits [91]. After dimerization of the subunits translocation occurs in the nucleus and NF-kB binds to DNA. Ag receptors, apoptosis, and host defence genes are regulated by NF-kB inside the nucleus. During immune response against pathogens, sensory and effector functions of TLRs are involved in the production of proinflammatory cytokines which ultimately increases the function of APCs, which have potential for immediate response against particular pathogen [92]. Differential activation of MAPK p38 and extracellular-signal-regulated kinase (ERK) within DCs results in altered levels of DC maturation and cytokine production. Studies have proved that activation of p38 has pivotal role in the DC maturation and proinflammatory immune responses and ERK activation has been much more needed for anti-inflammatory Th2 response [93, 94]. In SEA model, during infections, absence of TLR2 leads to enhanced the disease severity and Th1 and diminished Th2 responses [95]. DCs and B cells produce and activate IL-10 and TGF-*β* through MyD88 dependent pathway and the suppression of IL-12, IL-6, IL-1*β*, and TNF-*α* occurs by modulation of intracellular pathway of TLR2 by SEA [94].* Acanthocheilonema viteae*, a rodent nematode product of ES-62 glycoprotein, inhibits both B and T cell activation and TLR4 via MyD88 dependent pathway [70].

Clinically, for asymptomatic filarial infections, the deleterious pathology can be evaded by possible mechanism by downregulation of TLR on APC and T cells [96]. Previous literature shows TLR signaling in repose to intracellular pathogens including parasitic protozoa [97].* Wolbachia* extracts and* Wolbachia* surface protein (Wsp) can induce immune response through TLR2 and TLR4 [45, 46]. In mice model Hise et al. showed that Wsp induce an inflammatory response through TLR2 and TLR4 [98].* Wolbachia* mediated inflammatory responses mediated by TLR2 and TLR6 dependent on MyD88 and TIRAP/MAL. For intracellular parasite* Leishmania donovani* infection, the activation of TLR4 is mediated by MyD88 [99].

**Figure 1:**
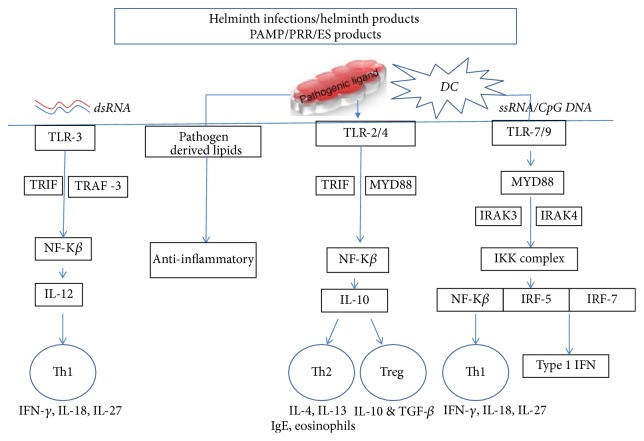
TLR interactions during helminth infections: depicting the involvement and induction of various TLRs during helminth infections and their byproducts. TLR pathways stimulation endorses specific Th environment.

TLR4 mediates accessibility to distinctive signaling pathway due to its cellular locations. Due to acidification of the endosome, this stimulates conformational changes that are the major properties to allow TLR4 differential use of adaptor proteins, involving distinct signaling pathways [100]. TLR8 recognizes viral ssRNA and endogenous RNA, such as microRNAs, resulting in the production of proinflammatory cytokines. Hence, localization sites of the receptors are crucial for the nucleic acid-sensing mode and downstream signaling [101]. B cell differentiation and activation of TLR signaling in B cells are initiated through B cell receptor or CD40 ligation. B regulatory cells are characterized by enhanced IL-10 production and in contrast downregulation of inflammatory reaction triggered by priming of B cell by TLR ligands [102]. The schematic diagram (Figure 1) depicts the involvement and induction of various TLRs during helminth infections and the byproducts.

## 7. Helminths and Immunomodulatory Sphingolipids

The human immune system can interact with carbohydrate, (glyco)protein, and lipid products of pathogens. Numerous studies of helminths have shown that one of the major properties of eukaryotic lipids is an immunomodulatory effect. One mechanism is by induction of Treg cells; Treg induction by a lipid product produced by schistosomes has been demonstrated [80]. Treg induction by* Schistosoma mansoni* egg antigens involves TLR2 expression. Lipids may also induce Th2 responses. Th2 development can be induced by diacylated phosphatidylserine, a lipid fraction of schistosomes that induces DC maturation [103]; this effect required TLR2 [80]. Similar findings have been reported in the immune response to* Ascaris lumbricoides*. Glycans may also play a role in resistance to* Schistosoma japonicum* [104]. In addition to TLR2, TLR9 may promote host protective immune responses. For example, TLR9 expression decreased the antileishmanial response by lipophosphoglycan (LPG) and TLR2.* Leishmania major* parasite infected with macrophages showed increased levels of LPG leading to decreased levels of TLR9 in comparison with a* Leishmania major* parasite with decreased levels of LPG. Study from Späth et al. demonstrated that* Leishmania* phosphoglycan^−/−^ cells were unable to persist in activated macrophages but recalled the ability to endure indefinitely in the mammalian host without provoking disease in nonactivated macrophages [105]. Activation of LPG helps in parasite survival in macrophages through TLR2 [106]. Thus, the induction of Tregs as well as other anti-inflammatory responses induced via interactions of a variety of helminth products with the innate immune system (APCs and iNKT cells) facilitates the survival of the helminth in the host and prevents inflammation.

## 8. Conclusion

TLRs provide a bridge between innate and acquired immunity. Moreover, TLRs not only are key players in the inflammatory process by promoting the production of inflammatory molecules, for example, cytokines and chemokines, but also function as regulatory (anti-inflammatory) contributors and appear to provide signals that are necessary for the resolution of excessive inflammation. In this review, we have explained how helminth-derived products, which provoke host responses, influence the immune system to prevent inflammatory diseases or immunopathology thus ensuring their survival in the host. One mechanism for the anti-inflammatory response induced by helminth-derived products is via their interaction with TLR 2/4 and TLR9. Sphingolipids and other lipids such as diacylated phosphatidylserine, glycans, and LPG lead to the induction of Th2 responses in helminth infection through stimulation of Tregs as well as other anti-inflammatory responses induced via interactions with the innate immune system enabling the survival of the helminth in the host and preventing inflammation. However, most of the factors that influence TLR induction of either proinflammatory or anti-inflammatory mediators are still to be elucidated. A further understanding of parasite derived TLR ligands can lead to innovative therapeutic and prophylactic strategies for parasitic infections.

## Additional Points

A comprehensive search approach using keyword and subject headings was applied to PubMed. One hundred and six articles were included. In addition, search engine searches using Google and Google Scholar were performed under the query “TLR and Helminth, TLR and signaling pathways, Immune responses to helminth and TLR, Innate immune responses”. The search was confined to articles written in English.
